# Universal iron supplementation: the best strategy to tackle childhood anaemia in malaria-endemic countries?

**DOI:** 10.12688/wellcomeopenres.19750.1

**Published:** 2023-08-16

**Authors:** Roshan Karthikappallil, Sarah H. Atkinson

**Affiliations:** 1 Department of Paediatrics, University of Oxford, Oxford, England, UK; 2 Centre for Tropical Medicine and Global Health, Nuffield Department of Medicine, University of Oxford, Oxford, England, UK; 3 Kenya Medical Research Institute (KEMRI) Centre for Geographic Medicine Coast, KEMRI-Wellcome Trust Research Programme, Kilifi, Kenya

**Keywords:** Malaria, Iron Deficiency, Iron, Anaemia, Children, Infant, Supplementation

## Abstract

Iron deficiency presents a major public health concern in many malaria-endemic regions, and both conditions affect young children most severely. Daily iron supplementation is the standard public health intervention recommended to alleviate rates of iron deficiency in children, but there is controversy over whether universal supplementation could increase the incidence and severity of malaria infection. Current evidence suggests that iron supplementation of deficient individuals is safe and effective in high-transmission settings when accompanied by malaria prevention strategies. However, low-resource settings often struggle to effectively control the spread of malaria, and it remains unclear whether supplementation of iron replete individuals could increase their risk of malaria and other infections. This review explores the evidence for and against universal iron supplementation programmes, and alternative strategies that could be used to alleviate iron deficiency in malaria-endemic areas, while minimising potential harm.

## Introduction

Iron deficiency (ID) is the most prevalent micronutrient deficiency worldwide, affecting over 1.2 billion people globally ^1^. It is the most common cause of anaemia and can cause severe and irreversible impairment of neurodevelopment in children ^2^. ID anaemia and malaria have significant geographical overlap, and both affect infants and preschool children most severely. Recent estimates suggest 64.1% of children aged 6–59 months in sub-Saharan Africa are anaemic ^3^, with over 60% of anaemia globally being caused by iron deficiency ^4^. Current World Health Organization (WHO) guidelines recommend that daily iron supplementation should be given to all infants and young children aged 6–24 months in settings where the prevalence of anaemia is 40% or higher in that age group ^5^. However, these guidelines are rarely implemented, partially due to controversy over whether iron supplementation may increase infection and severity of malaria, particularly in iron replete children. Untangling the complex and controversial relationship between iron and malaria is vital to evaluate the safety and effectiveness of universal iron supplementation in malaria-endemic regions.

## Why is childhood iron deficiency prevalent in low-resource settings?

### Diet

The main driver of iron deficiency in low-resource settings is a lack of bioavailable iron in staple foods ^6^. As animal (heme) sources of iron have higher bioavailability than vegetable sources, invariable plant-based diets prevalent in low-resource settings can predispose populations to ID anaemia. For example, a cross-sectional study of 6 to 10-month-old infants in southern Kenya found that 52.2% consumed only maize porridge as their main complementary food, with only 9% consuming any meat ^7^. Diets are often also low in fruit intake (which can enhance iron absorption) and high in cereals (many of which contain phytate that hinders absorption) ^8^.

### Infection

Hepcidin is a hormone that reduces absorption of iron from the gut lumen and serves as the primary mechanism by which the body regulates iron levels. Hepcidin is secreted by the liver as an innate response to infection and works to restrict iron supply to pathogens. As infection reduces absorption of dietary iron *via* hepcidin, high rates of respiratory ^9^ and malaria infections ^10^ can also contribute towards ID rates. Parasitic infection with whipworm and hookworm can cause chronic intestinal blood loss, increasing iron demand. A meta-analysis of hookworm infection found that both low-intensity and high-intensity infection were significantly associated with a reduction in haemoglobin levels in school-age children ^11^. The burden of infection is an important contributor to childhood iron deficiency in low-resource settings.

### Growth

Young children are particularly susceptible to ID anaemia, due to their high iron requirements during growth and development. From around 4–12 months of age, children are in a period of ‘iron famine’ as their blood volume rapidly expands with growth, and high rates of erythropoiesis are required to maintain haemoglobin concentration ^12^. Longitudinal studies have demonstrated that hepcidin levels are highest in young infants, reducing their ability to effectively absorb iron from the diet ^13^, and so they are dependent on iron stores from pregnancy. The high prevalence of maternal ID in low-resource settings reduces the size of these neonatal iron stores, further exacerbating the problem of childhood ID ^14^. Nevertheless, a longitudinal analysis in Gambian infants found that weight gain correlated with decreasing hepcidin alongside decreasing ferritin levels (indicating increased iron absorption alongside depleted iron stores) ^15^, suggesting that while rapidly growing infants are the most susceptible population to iron deficiency, they are also the group most likely to be responsive to oral iron supplementation. High iron demand combined with poor iron availability in the diet makes early childhood the highest risk period for iron deficiency and iron deficiency anaemia, and it can be almost impossible for resource-constrained households to provide adequate iron without supplementation.

**Figure 1.**
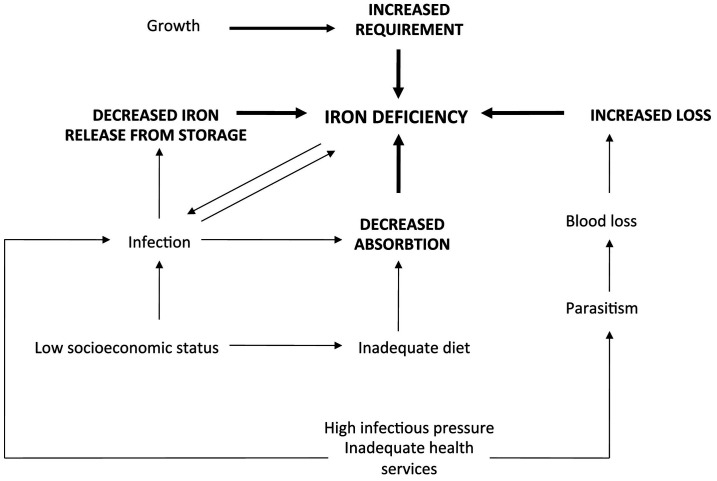
Causes of iron deficiency in children living in poorer countries. Adapted from Jonker *et al.*, 2017 ^16^.

The causes of iron deficiency in low-resource settings are summarised in Figure 1 ^16^.

## Difficulties in assessing iron deficiency

There is significant controversy over the effectiveness of current iron markers and established thresholds for the diagnosis of ID, as many iron markers are confounded by infection or inflammation and cannot be easily normalised by comparison with markers of inflammation ^17^. The most used iron markers include haemoglobin and serum ferritin levels, but each of these has its own caveats.

### Use of haemoglobin as an iron marker

While haemoglobin levels are very easy to measure, anaemia (defined by WHO as a haemoglobin level of less than 110 g/L) ^18^ is not specific to iron deficiency. As malaria itself can cause anaemia, haemoglobin is generally unsuitable to measure iron status in malaria-endemic areas unless used in conjunction with other iron markers. In addition, evidence from human studies ^19^ and animal models ^20, 21 ^shows that the brain’s iron status is compromised before that of red blood cells, suggesting that children who are iron deficient without anaemia may still experience the neurodevelopmental defects associated with iron deficiency.

### Use of serum ferritin as an iron marker

Serum ferritin is the WHO recommended marker of iron status, and is accurate in healthy individuals, but upregulated in infection due to its role as an acute phase reactant. Therefore, use of serum ferritin as a measure of iron deficiency will underestimate the prevalence of ID in areas where infection is prevalent ( *i.e.*, malaria-endemic regions). The cut-offs used by WHO to define ID have been updated to include increased cut-offs in the presence of infection ( Table 1) ^22– 24^.

**Table 1.** Recommended serum ferritin cut-off values to define iron deficiency in apparently healthy and non-healthy children by age group ^24^.

Age group	Serum ferritin (μg/L)	
	Apparently healthy individuals	Individuals with infection or inflammation
Infants and young children (0–23 months old)	<12	<30
Children under 5 years (24–59 months old)	<12	<30
Children (5 to less than 10 years old)	<15	<70

Rather than the use of an arbitrarily raised cut-off for serum ferritin in the presence of inflammation, it has been suggested to use an internal linear regression to correct iron marker levels for malaria parasitemia and for inflammation using C-reactive protein (CRP) or α1-acid glycoprotein (AGP) measurements ^25^. A comparison by Muriuki *et al.*, (2020) between the current WHO guidelines and a proposed internal regression definition found that the WHO guidelines significantly underestimated the prevalence of ID in African children across four countries (34% with the WHO definition compared to 52% using the internal regression estimate) ^26^. Nevertheless, Castberg *et al.*, measured conventional iron markers in children recovering from acute malaria episodes and found that inflammatory markers normalise at least two weeks earlier than serum ferritin ^17^. This means that techniques used to correct serum ferritin and other iron markers for inflammation may still be insufficient to eliminate bias in studies carried out in malaria-endemic areas. This also suggests that targeted supplementation strategies ( *i.e.*, where iron supplementation is only given to those who are shown to be iron deficient) risk underestimating the prevalence of ID in malaria-endemic areas, leading to higher rates of anaemia.

The Biomarkers Reflecting Inflammation and Nutrition Determinants of Anemia (BRINDA) project was set up in 2012 with the aim of improving micronutrient assessment and anaemia characterisation globally. The BRINDA project pooled national and regional nutritional surveys over the previous 10 years representing ~30,000 preschool children and 21,000 school age children from all six WHO geographic regions, and measured haemoglobin and CRP levels alongside at least one other indicator of iron status ( *e.g.*, serum ferritin or serum transferrin) ^27^. They found that subclinical inflammation (as indicated by acute phase reactant measurements) is prevalent even in low-infection-burden countries such as the United States, and that low levels of inflammation can bias iron status indicators ^28^. Therefore, the problem of estimating the ‘true’ burden of iron deficiency and iron deficiency anaemia rates is not restricted to high-infection-burden countries.

### Use of serum transferrin saturation as an iron marker

Transferrin saturation has been found to be less affected than serum ferritin by inflammation and malaria parasitaemia when tested against other iron markers ^26^ (including iron, transferrin levels, hepcidin, haemoglobin, and serum transferrin receptor) and was found to most closely match the current gold standard for iron deficiency measurement (regression corrected ferritin levels). However, despite this, the area under the curve for transferrin saturation was only 0.77, suggesting limited sensitivity as a diagnostic tool.

### Hepcidin as an indicator of iron need

Potential solutions to this problem may include combining multiple iron biomarkers or switching the focus from biomarkers of iron levels to biomarkers of iron need. Recently, hepcidin itself has attracted attention as a clinically useful indicator for iron supplementation. Hepcidin is the key iron regulator in the body and is downregulated in iron deficient populations to promote iron absorption but upregulated during infection in order to restrict iron supply to pathogens. Therefore, high hepcidin levels indicate either that the child is iron replete, or that they currently have low iron absorption rates due to infection or inflammation—both of which would indicate that iron supplementation would be ineffective. Prentice *et al.*, used a univariate statistical model to compare multiple iron markers as predictors of iron incorporation into erythrocytes after supplementation, and found that hepcidin was the most accurate determinant of iron incorporation over 30 days, providing further evidence that the development of cost-effective assays for hepcidin could prove valuable in iron supplementation trials ^29^.

## What are the benefits of universal iron supplementation in children?

### Child development and growth

Iron plays key roles in oxygen transport and cellular metabolism and is essential for immune and neural development. Iron deficiency may have deleterious effects on neurodevelopment in children: directly through effects on iron-dependent myelination and long lasting epigenetic alterations to metabolism ^20^, and indirectly through ID anaemia, which can cause impaired cognitive and motor development, reduced growth velocity, and anorexia ^30^. Of particular concern are studies that indicate that these changes may lead to long-term deficits in executive function and recognition memory ^2^. Even iron deficiency that is not severe enough to cause anaemia (and therefore not detected by common screening procedures), has been found to adversely affect infant social-emotional behaviour at 12 months ^31^. Iron supplementation in at-risk populations during the first year of life has been shown to improve motor outcomes, neurocognitive and language outcomes, and social development ^32^, and the opportunity to avoid these knock-on effects in development has made establishing iron sufficiency in children a key public health goal worldwide.

However, while meta-analyses have shown that iron supplementation programmes are effective at reducing rates of anaemia, they are equivocal about impact on functional outcomes ^33^. Indeed, a randomised controlled trial by Pasricha *et al.*, (2021) found that while both oral iron supplementation and iron-containing micronutrient powders decreased the prevalence of anaemia in Bangladeshi infants compared to a placebo, there was no difference in cognitive development, or on any other developmental or growth outcome between treatment groups and the placebo group, either immediately after the three month regimen or at follow-up nine months later ^34^.

There is also some concern that iron supplementation in children who are iron replete may cause harm to development. A Chilean study found a neurodevelopmental benefit at 12 months in infants given high-iron formula feed ^35^, however children who had a high 6-month haemoglobin (>128 g/L) and were given high-iron feed had poorer neurodevelopmental scores at 10 years old when compared to the low-iron group ^36^. A potential explanation for this could be that children who have high iron levels at baseline ( *i.e.*, due to genetic factors) may be adversely affected by iron fortification during early life, which would suggest caution when it comes to universal supplementation programmes. Nevertheless, the authors also note that high Hb levels do not necessarily reflect iron status and could instead be a marker of chronic hypoxia due to another factor, such as maternal smoking, which itself is linked to poor neurodevelopment in children ^37^. Indeed, a later study by Amin *et al.*, (2012) found no increase in neurodevelopmental impairment at 8–12 months in premature infants with iron overload compared to premature infants with normal iron status, after controlling for confounders ^38^. While more research is needed in this area, there is very little evidence to suggest a major risk of iron overload due to universal iron supplementation programmes.

### Immunity

Iron is essential to maintaining immunity, with ID anaemia associated with significantly lower baseline IgG levels, reduced IL-2 and IL-6 production, and reduced phagocytic activity ^39^. Iron acts as a co-factor for the production of enzymes such as myeloperoxidase and nitric oxide synthase that are required for the killing of intracellular pathogens ^40^, with iron deficiency being shown to both blunt neutrophil production and suppress antibacterial mechanisms in neutrophils ^41^. Iron deficiency has been linked to a reduced immune response against specific pathogens, including bacterial infections ^42^ and malaria ^43^. A prospective study into the effect of ID anaemia on infection amongst Bedouin infants found that anaemia at six months old was an independent risk factor for diarrhoea and respiratory disease from 7–18 months of age, even after controlling for environmental and socio-economic factors ^44^. Iron deficiency can therefore be both a cause and consequence of infection. This positive feedback effect creates a vicious cycle that results in high healthcare burdens for low-income countries.

Studies into the effect of iron on adaptive immunity show similar trends. A case control study of 40 children with ID anaemia *vs.* 40 age-matched healthy children found that the ID anaemia group had significantly lower CD4+ T cell counts and decreased CD4:CD8 ratios. Oral iron supplementation was found to significantly improve CD4+ T cell counts and CD4:CD8 ratios in the ID anaemia group ^41^. A recent cohort study in Kenyan infants also suggests that ID anaemia may reduce antibody responses to vaccines, with a follow-up randomised controlled trial (RCT) specifically investigating the response to the measles vaccine showing that iron supplementation prior to vaccination helps to restore the humoral response ^45^. These findings have significant impact for public health and policy, as they indicate that correcting childhood iron deficiency may be a cost effective and clinically efficacious method of combating infectious disease.

## What are the risks of universal iron supplementation?

### Malaria

In 2006, a large prospective randomised control trial (RCT) conducted on Pemba Island, Zanzibar (hereafter known as the Pemba Trial) was stopped prematurely because both arms receiving iron and folate supplementation had a higher rate of adverse events. In particular, malaria-related hospital admissions were significantly higher in the iron arms than in the placebo arm (R, 1.18; 95% confidence interval [CI] [1.02, 1.36]) ^46^. A similar trial conducted in Nepal (a non-malarious area), however, found no evidence of increased mortality in the iron arm compared to controls ^47^. The results of these trials raised concerns over whether universal iron supplementation was safe in malaria-endemic regions.

### How could host iron status increase malaria risk?

Several mechanisms have been proposed to explain this association. *Plasmodium*, the group of parasites that cause malaria, are dependent on iron both in the clinically silent hepatocyte stage and disease associated erythrocyte stage to proliferate. Clark *et al.*, (2015) established a potential causal mechanism for the effects of iron on malaria risk, showing that malaria growth is reduced in red blood cells (RBCs) from individuals with ID anaemia and increased in RBCs from iron-supplemented donors ^48^. Iron supplementation may also increase the amount of non-transferrin-bound iron (NTBI), which promotes free radical formation and is more bioavailable to *Plasmodium*. Alternatively, iron’s effect on malaria may be mediated *via* the immune system, rather than directly through effects on *Plasmodium*. For example, infected RBCs are easier to phagocytose in iron-deficient hosts ^49^, increasing the ability of the immune system to control and neutralise infections. It is likely that all these mechanisms contribute to the effect of host iron status on malaria.

### Does the evidence base support the iron supplementation-malaria hypothesis?

Iron has complex effects on immunity and host-pathogen interactions, and iron supplementation may increase the risk of infectious disease. Several observational studies have demonstrated that iron deficient children have a reduced risk of malaria, even accounting for misclassification and other potential causes of bias ^50, 51^. For example, a prospective cohort study by Muriuki *et al.*, (2019) found that iron deficient children had a malaria incidence rate ratio of 0.7 compared to iron replete children (95% CI [0.6, 0.8]; P<0.001) over a six month period ^52^. Similarly, high iron levels have been associated with an increased risk of malaria, with Moya-Alvarez *et al.*, (2017) finding that infant iron levels were significantly associated with risk of a positive blood smear (adjusted odds ratio, 2.90; 95% CI [1.68, 4.98]; P<0.001) and *Plasmodium falciparum* parasite density during the first year of life ^53^.

Although observational studies have associated host iron status with increased risk of malaria, RCTs into the safety of oral iron supplementation (including a sub study of the original Pemba trial ^46^) have not detected any additional risk when used in conjunction with appropriate malaria management and prevention strategies ^54^. The most recent Cochrane review into the safety of iron supplementation in malaria-endemic areas analysed 35 individually and cluster RCTs conducted in areas where malaria was hyper- or holo-endemic (prevalence over 50 or 75%), with evidence from 31,955 children aged 0–18 years ^55^. It found that overall, iron does not cause an excess of clinical malaria in the presence of effective malaria control (risk ratio (RR), 0.93; 95% CI [0.87, 1.00]). In areas without prevention and management programmes for malaria, iron may increase malaria risk (RR, 1.16; 95% CI [1.02, 1.31]). Based on this evidence, WHO changed their guidelines in 2016 to recommend universal iron supplementation in malaria-endemic settings “in conjunction with public health measures to prevent, diagnose, and treat malaria” ^56^.

However, the 2016 Neuberger *et al.*, Cochrane review has its own limitations ^55^. Data on children’s baseline iron status were not collected in the trials analysed, and so the authors were unable to carry out subgroup analysis of the effect of iron supplementation on iron deficient *vs.* iron replete children. This is an important limitation, as the Pemba trial sub study showed that iron supplementation benefited iron deficient children, reducing the rate of adverse events by 38% (p=0.02), but may have harmed iron replete children, increasing rate of adverse events by 63% (p=0.24) ^46^. While this effect was not significant in the Pemba sub study, the lack of baseline iron status measurement in studies used in the Neuberger meta-analysis means it is impossible to tell if harm done to iron replete children was masked by the benefit to iron deficient children.

It is also important to consider whether the trials included in the 2016 Cochrane Review had an appropriate trial outcome. For example, the Pemba trial only reported on malaria that resulted in hospital admission, and therefore there is a risk that it underestimated the total incidence of malaria in the trial population. All other trials used clinical malaria as an endpoint (defined as fever accompanied by parasitaemia). However, this endpoint excludes asymptomatic parasitaemia, which is common in areas with high rates of malaria transmission. Therefore, it is possible that the trials included in the meta-analysis systematically underestimated the prevalence of malaria infection, reducing the strength of the association between iron supplementation and malaria.

The authors also note that results from cluster RCTs may not have been appropriately adjusted, which may have had a significant impact on the results of the meta-analysis. Nine out of the 35 trials included were cluster RCTs, and as the largest trials they carried significant weight in the meta-analysis. Six out of nine of these cluster RCTs failed to report on the intracluster correlation coefficient (denoting degree of similarity in variables such as iron status for children in the cluster). The authors had to estimate intracluster correlation coefficients to adjust the weight of the cluster RCTs in meta-analysis, which reduces the quality of the conclusions reached.

While RCTs are treated as the gold standard in epidemiology, they are not without their flaws. Individuals in RCTs are subject to active surveillance and are therefore more likely to receive prompt treatment in the case of adverse events. In addition, RCTs measure only a limited duration of iron supplementation and may not be representative of the child population in need of iron supplementation. While efforts can be made to make RCTs more realistic, they will never be able to replicate the epidemiological reality of malaria-endemic settings as observational studies do.

Overall, while the evidence suggests that iron supplementation is safe in malaria-endemic settings when accompanied by malaria prevention and management strategies, areas of uncertainty remain. These include whether iron supplementation increases malaria risk in iron replete children, or in low-transmission areas, and what levels of malaria prevention and management are sufficient to justify the safe use of universal iron supplementation strategies.

### Gut microbiome

Iron supplementation may also alter the gut microbiome, increasing the risk of gram-negative bacterial infection and bloody diarrhoea in children. A cluster RCT by Soofi *et al.*, (2013) examined the safety of micronutrient powders (MNPs) in fortifying the iron content of home meals over 12 months and found that both micronutrient groups had higher rates of bloody diarrhoea and higher overall morbidity than the unfortified group ^57^. This study was limited by its lack of a placebo control group, and it is unclear how translatable this trend is to other oral iron supplementation programmes. However, a range of *in vitro* and *in vivo* studies suggest that both MNP fortification and other forms of oral iron supplementation significantly increase colonic iron levels, and adversely alter the gut microbiome by decreasing the balance of commensals to enteropathogenic pathogens, resulting in gut inflammation and increased risk of diarrhoea ^58^. This is an important side effect to note, as diarrhoea is a significant contributor to infant mortality in low-resource settings and is estimated to contribute to the deaths of approximately one in nine children less than five years old in sub-Saharan Africa ^59^.

### Reactive oxygen species

Non-transferrin bound iron has the potential to react with oxygen to create reactive oxygen species (ROS) that can damage lipid membranes within the body. This is a risk primarily in children with low concentrations of iron-binding proteins whose systems are overwhelmed by high dose iron supplementation therapies. For example, malnourished children with kwashiorkor have lower concentrations of transferrin, and this is associated with increased mortality ^60^. It is therefore possible that iron supplementation in malnourished paediatric populations may carry increased risk. Nevertheless, at present, there is very little clinical evidence of iron supplementation causing increased oxidative stress in children. Indeed, a prospective observational study in which 21 healthy six-week infants with very low birth weight were given high dose iron (18 mg daily) found no significant change in markers of oxidative stress in the urine. This suggests that any additional ROS production caused by iron supplementation is clinically insignificant. However, the major caveat to this study is that iron supplementation was only given for one week, which may not have been a long enough period for markers of oxidative stress to increase significantly. Additionally, the study only observed a small sample of healthy premature infants, and so it is unknown how well these findings translate to a larger population of children from a wide age range who may suffer from infection or malnutrition ^61^.

## How can we implement iron supplementation as safely as possible?

Current evidence suggests that iron supplementation may still carry risks in low-income areas where malaria control is ineffective and the burden of infectious disease is high. Given this, what practical strategies are there to balance the safety and efficacy of iron supplementation strategies in children?

### Adjusting the timing of iron supplementation

Atkinson *et al.*, (2014) analysed iron status and hepcidin levels over the course of a malaria season in The Gambia and Kenya and found that ID increased and hepcidin levels decreased over the course of the malaria season ^62^. A more recent study by Roberts SA *et al.*, (2021) found the same association in adolescents in Burkina Faso, with higher hepcidin concentrations in the wet season corresponding with rising malaria prevalence ^63^. Therefore, targeting iron supplementation programmes at the end of the malaria or wet season could maximise their effectiveness (as low hepcidin levels mean iron absorption is at its peak), target the populations that need it the most (as ID is most prevalent at this time), and increase their safety (as malaria rates are lower).

### Food fortification

An alternative strategy to boost iron intake at a population level is food fortification. Several methods can be used to fortify food with iron, including biofortification of staple food crops, industrial fortification ( *i.e.*, fortification of staple foods at the point of manufacture, such as fortified wheat), and point-of-use fortification ( *i.e.*, addition of a micronutrient powder to meals, MNP, or small quantity lipid based nutrient supplements, SQ-LNSs ^64^). These strategies enable the delivery of small doses of iron several times a day, which, while slower to raise iron levels, may prove safer than oral supplementation. Biofortification and industrial fortification are effective in malaria-endemic countries ^65^ and have several practical advantages over supplementation, in that they reach all sections of society, are not dependent on patient compliance, and have lower costs than oral iron supplementation programmes ^66^. Point of care fortification with MNPs and SQ-LNSs still require uptake by the population but could prove safer and milder in side-effect profile than oral iron supplementation. However, the effectiveness of food fortification strategies in reducing rates of ID anaemia is dependent on a host of contextual factors, including cooking techniques, staple foods (and whether they aid or counter iron absorption) and the chemical composition of MNPs/SQ-LNSs, leading to variability in outcomes between countries ^67^. For example, a MNP intervention in Uganda by Ford *et al.*, 2020 found no change in anaemia status despite high programme fidelity, and theorised that cooking with soda ash, which reduces micronutrient bioavailability and absorption, may explain the lack of effectiveness ^68^.

### Targeted supplementation and the difficulties of assessing host iron status

The results of the Pemba trial in 2006 caused WHO to adjust its guidelines that same year, recommending that iron supplementation be given to children only after screening for iron deficiency ^69^. This has the benefit of avoiding any potential harm to iron replete children. However, targeted supplementation strategies have their own drawbacks, such as the significant economic and logistical burden of screening whole populations for ID and anaemia, difficulty assessing iron status adequately in malaria-endemic regions, and the risk of under-estimating and under-treating iron deficiency in the population. These challenges, alongside accumulating evidence from RCTs that the effect of iron supplementation on malaria risk was overstated, led WHO to revert their guidelines in 2016 to recommend universal supplementation but with effective malaria control ^5^.

### Alternatives to iron supplementation

Other approaches involve tackling the causal factors of iron deficiency at a population level. As infants in low-resource settings often have insufficient iron stores from pregnancy, delayed umbilical cord clamping after birth has been suggested as a method to increase fetoplacental blood transfusion and infant blood volume. This strategy has been shown to increase infant iron stores and prevent ID in early infancy, with an RCT demonstrating that delayed clamping for >3 minutes after birth reduces the relative risk of anaemia for up to 12 months (RR, 0.91; 95% CI [0.84, 0.98]) ^70^. Infectious disease and helminth infections are another significant cause of iron deficiency at a population level, and so deworming programmes are another important avenue to prevent ID, with a paper by Byrne *et al.*, (2021) demonstrating that deworming using albendazole is associated with significantly raised haemoglobin levels in children ^11^. A growing evidence base suggests that malaria contributes to the burden of iron deficiency, with a recent paper by Ntenda *et al.*, (2022) concluding that both asymptomatic and clinical malaria were independent risk factors for anaemia and functional iron deficiency (FID) ^71^. Therefore, malaria elimination programmes themselves may be an effective strategy to address ID in malaria-endemic areas ^10^. For example, Frosch *et al.*, noted significant increases in haemoglobin and serum ferritin levels in children <5 years old after a 12-month period of interrupted malaria transmission in highland Kenya ^72^.

## Conclusions

Current evidence suggests that universal iron supplementation is safe in malaria-endemic areas when accompanied by appropriate malaria prevention and diagnosis. In low-resource settings where malaria control is inadequate, there is some evidence that iron supplementation may increase the risk of malaria and gram-negative bacterial infection in children. On the balance of risks, it is likely that the benefits of universal iron supplementation in reducing population levels of iron deficiency and anaemia outweigh any potential increases in the risk of infection.

The current literature base is limited by a lack of accurate iron markers that are unbiased by the high rate of infection in malaria-endemic areas, and so new markers must be developed to investigate the effects of host iron status on the safety of iron supplementation. From a health policy perspective, addressing the causal factors of iron deficiency could reduce the need for iron supplementation, while in the meantime, small changes to iron supplementation programmes (such as aiming them at the end of the wet season) could increase their efficacy and limit any potential risks.

Looking to the future, thought must also be given to the changing public health landscape of malaria endemic areas. Many people living in malaria-endemic areas are currently undergoing rapid dietary and lifestyle changes associated with increased urbanisation. While lifestyle diseases are often seen as solely the problem of high-income countries, urban settings in Sub-Saharan Africa and South America are increasingly facing ‘the dual burden of disease’, in which conditions such as iron deficiency anaemia and obesity present in the same children. A recent study of children in Lima, Peru, showed that both adiposity and CRP were associated with a lack of response to iron supplementation after one month of treatment ^73^. Therefore, sufficient attention must be given to ensure that childhood obesity and the chronic inflammation associated with it does not exacerbate the challenges associated with treating iron deficiency in malaria-endemic areas.

## Data Availability

No data are associated with this article.
