# Supplementary material for: Universal iron supplementation: the best strategy to tackle childhood anaemia in malaria-endemic countries?
Source: Wellcome Open Res. 2023 Aug 16;8:345. [Version 1] doi: 10.12688/wellcomeopenres.19750.1 (PMC10541535; doi:10.12688/wellcomeopenres.19750.1)
Supplement: Peer review history

# Reviewer report: Reviewer response for version 1

**DOI:** 10.21956/wellcomeopenres.21876.r66436

**Published:** 2023-09-29

**Authors:** Raffaella Gozzelino

**Affiliations:** 1 Universidade NOVA de Lisboa, Lisbon, Portugal

In this article, Abuga K.M. et al describe the impact of severe anemia on bacterial infections, a scenario that is recurrent in Saharan Africa, where this association constitutes a major health concern. Prevalent pathogens and risk factors for specific comorbidities are reported, as the importance of iron metabolism in activating the innate and adaptive immunity by discriminating its function between different immune cells. This comprehensive review also highlights the role of this metal in ensuring the survival of both pathogen and host, emphasizing the need for additional studies to better address the effects of severe anemia on vaccine efficacy.

 The article is well written and the authors provide an interesting overview on the current knowledge on this field.

Is the review written in accessible language?

Yes

Are all factual statements correct and adequately supported by citations?

Yes

Are the conclusions drawn appropriate in the context of the current research literature?

Yes

Is the topic of the review discussed comprehensively in the context of the current literature?

Yes

Reviewer Expertise:

Immunology, infectious diseases, malaria, neurodegeneration, neurodegenerative diseases, African countries

I confirm that I have read this submission and believe that I have an appropriate level of expertise to confirm that it is of an acceptable scientific standard.

# Reviewer report: Reviewer response for version 1

**DOI:** 10.21956/wellcomeopenres.21876.r66440

**Published:** 2023-09-26

**Authors:** Elpis Mantadakis

**Affiliations:** 1 Department of Pediatrics, Democritus University of Thrace Medical School, Thrace, Greece

The manuscript “ ***Universal iron supplementation: the best strategy to tackle childhood anaemia in malaria-endemic countries?”*** by Karthikappallil and Atkinson is a well-written and adequately referenced review of universal iron supplementation strategies in malaria-endemic countries to combat iron deficiency anemia. I would only expand on recent knowledge regarding the risks of using iron supplements for improving iron deficiency in infants (Zimmermann MB. Global look at nutritional and functional iron deficiency in infancy. *Hematology Am Soc Hematol Educ Program* 2020;2020(1):471-477). Although iron repletion increases anti-measles immunoglobulin G seroconversion, a very useful effect in low-income settings, unabsorbed iron reaching the colon adversely affects the gut microbiome causing gut inflammation and diarrhea. To this extent, galactoligosaccharides and apolactoferrin may prove useful in malaria-endemic areas by increasing the absorption of iron and simultaneously lessening the adverse effects of unabsorbed iron on the infant’s gut.

Is the review written in accessible language?

Yes

Are all factual statements correct and adequately supported by citations?

Yes

Are the conclusions drawn appropriate in the context of the current research literature?

Yes

Is the topic of the review discussed comprehensively in the context of the current literature?

Yes

Reviewer Expertise:

Benign Pediatric Hematology Oncology, iron deficiency prevention and treatment, parenteral iron therapy

I confirm that I have read this submission and believe that I have an appropriate level of expertise to confirm that it is of an acceptable scientific standard.

## References

[1] : Global look at nutritional and functional iron deficiency in infancy. *Hematology Am Soc Hematol Educ Program* .2020;2020(1) : 10.1182/hematology.2020000131 471-477 10.1182/hematology.2020000131 33275751PMC7727574
